# Effects of Temperature, Ionic Strength and Humic Acid on the Transport of Graphene Oxide Nanoparticles in Geosynthetic Clay Liner

**DOI:** 10.3390/ma17092082

**Published:** 2024-04-28

**Authors:** Yaohui Liu, Tao Jiang

**Affiliations:** 1 School of Mechanics and Civil Engineering, China University of Mining and Technology, Xuzhou 221000, China; 2 Department of Geotechnical Engineering, College of Civil Engineering, Tongji University, Shanghai 200092, China; 1710290@tongji.edu.cn

**Keywords:** transport, graphene oxide, geosynthetic clay liner, temperature, deposition

## Abstract

With the wide application of graphene oxide nanoparticles (GONPs), a great amount of GONP waste is discarded and concentrated in landfills. It has been proven that GONPs have strong toxicity and could gather toxic substances due to their high adsorption capacity. GONPs will seriously pollute the surrounding environment if they leak through the geosynthetic clay liner (GCL) in landfills. To investigate various factors (temperature, ionic strength (IS) and humic acid (HA)) on the transport and retention of GONPs in the GCL, a self-designed apparatus was created and column tests were carried out. The experimental results show that GONPs could be transported through the GCL. The mobility and sorption ratio of GONPs in GCL decreased with an increase in temperature and IS, and increased with an increase in HA. The temperature had little effect on the deposition ratio of GONPs in the GCL. The deposition ratio of GONPs in the GCL increased with IS, and decreased with an increase in HA. The transport of GONPs in GCL, glass beads and quartz sand was compared, and the results show that the retention ability of the GCL is much better than other porous materials. The experimental results could provide significant references for the pollution treatment in landfills.

## 1. Introduction

With the wide application of nanometer materials, increasing nanometer wastes are produced. Reports show that the total output of nanometer materials in the world is 260,000~309,000 tons, and 63~91% of these materials are discarded in landfills [1]. Previous studies have proven that nanoparticles pose a serious risk to the environment [2,3,4]. Barlaz et al. [5] and Khan et al. [6] found that nanoparticles could be transported into the interior of landfills, and this aggravated the pollution of nanometer particles.

Graphene oxide nanoparticles (GONPs) are one of the most widely used nanoparticles. Kamenska et al. [7] investigated the impact of polyethylene glycol functionalization of graphene oxide on anticoagulation and the hemolytic properties of human blood. Li et al. [8] investigated the application of graphene oxide composite catalysts for high-efficiency and bifunctional catalysts for photocatalytic dye degradation and hydrogenation. A great number of GONPs were released into the environment during the industrial cycle and finally concentrated in landfills [9,10]. GONPs have been proven to have strong toxicity [11,12] and adsorption capacity [13] and thereby could gather toxic substances in landfills. Dibyanshu et al. [14] investigated the possibility of GONPs becoming an emerging contaminant in groundwater and proposed the corresponding treatment strategy. Additionally, the mobility of GONPs was greater than that of other nanoparticles due to their high water solubility and stability, resulting in the serious harm of GONP leakage into landfills [15]. Therefore, it is crucial to investigate the transport and leakage of GONPs in landfills.

Many researchers have investigated the transport of GONPs in porous media [16,17,18]. Many factors, e.g., humic acid (HA) [19,20], ionic strength (IS) [21] and pH [22,23], have been proven to have an influence on the transport of GONPs in porous media. Cao et al. [24] proved that the mobility of GONPs is highly sensitive to ionic strength and pH. Wang et al. [25] investigated the co-transport of GONPs and hematite colloids (a model representative of iron oxides) in saturated sand through column experiments. The results demonstrated that the presence of hematite colloids inhibited GONP transport in quartz sand columns. The temperature inside the landfill is significantly higher than the normal temperature in nature. This is because the chemical substances inside the landfill will react to produce heat. Additionally, heat is difficult to emit because the landfill is buried underground. High temperatures may have a serious impact on the migration of GONPs. However, studies on the effect of temperature on the transport of GONPs are still rare.

The transport of GONPs in various porous media such as silica sand [26,27], glass bead [28] and loess [29] has been observed by many researchers, and the results showed that the types of media have a significant influence on the transport of GONPs. Chen et al. [30] investigated the effects of low-molecular-weight organic acids (LMWOAs) on the transport of GONPs in saturated kaolinite- and goethite-coated sand columns. Esfahani et al. [31] evaluated the effect of co-transport of different-sized microorganisms on graphene oxide nanoparticle (GONP) transport and the retention in saturated pristine and biofilm-conditioned limestone columns. However, the above studies were based on the transport of GONPs in traditional porous media. The geosynthetic clay liner (GCL) is one of the most important barriers preventing the leakage of pollutants in landfills [32,33,34]. Previous studies have shown that the GCL can reduce the ground subsidence caused by garbage leakage, improve the stability of landfills and prolong the service life of landfills [35,36]. One of the main components of the GCL is bentonite particles, and the adsorption of bentonite particles is pretty large, resulting in the fact that the retention mechanism of the GCL is quite different from traditional porous media [37]. However, investigations on the transport of GONPs in the GCL are still ongoing. It is still unclear if GONPs could be transported through the GCL and what the transport behavior of the GONPs in the GCL is like under different conditions.

To investigate the transport and retention rule of GONPs in the GCL, flexible wall permeameters were modified and column tests were carried out. The influence of various factors (temperature, IS and HA) on the transport of GONPs was investigated and analyzed. The transport of GONPs in different media was compared. The experimental results can provide some technical references for the prevention of GONP pollution.

## 2. Materials and Methods

### 2.1. GONPs

GONPs were derived from Suzhou Heng-qiu Company (Shanghai, China). GONPs are produced through pressurized oxidation method, and the specific manufacture method has been recorded by many investigations. The characteristics of GONPs are summarized in Table 1.

### 2.2. Geosynthetic Clay Liner

**Figure 1:**
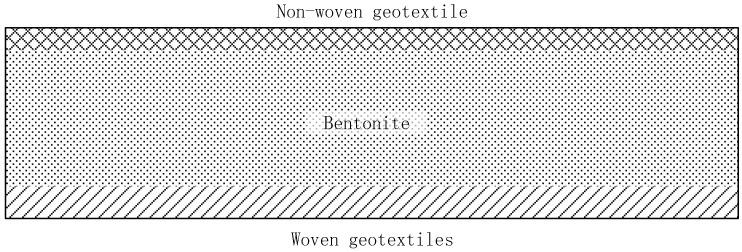
Structure of the GCL.

The GCL was prepared through stitching sodium bentonite particles between two layers of geotextiles, as shown in Figure 1. According to the information provided by manufacturer, the bottom layer was woven geotextile with a unit mass of 221 g/cm^3^, and the top layer was non-woven geotextile with a unit mass of 112 g/cm^3^.

### 2.3. Concentration Determination of GONPs

The concentration of GONPs is difficult to determine directly at present. The most widely used method to determine the GONP concentration is the indirect method of ultraviolet–visible spectrophotometry (UV-vis). However, the type and size of GONPs may have an influence on the measuring results. Therefore, calibration tests for UV-vis were carried out, and the results can provide some references for other scholars. Previous investigations have proven that the characteristic adsorption wavelength of GONPs was 230 nm [38]. The relationship between GONP concentration and absorbance was investigated before column tests. GONP suspension (500 mL) was shaken through ultrasonic oscillator for 30 min. Then, suspension was diluted through deionized water. According to the chemical analysis of leachate in landfills, the final concentration range of GONPs was determined from 5 mg/L to 30 mg/L. Suspension was shaken through ultrasonic oscillator for 30 min again, and the absorbance of wave was measured through UV-vis [38]. The experiment was repeated three times, and the average value was taken.

### 2.4. Experimental Setup

**Figure 2:**
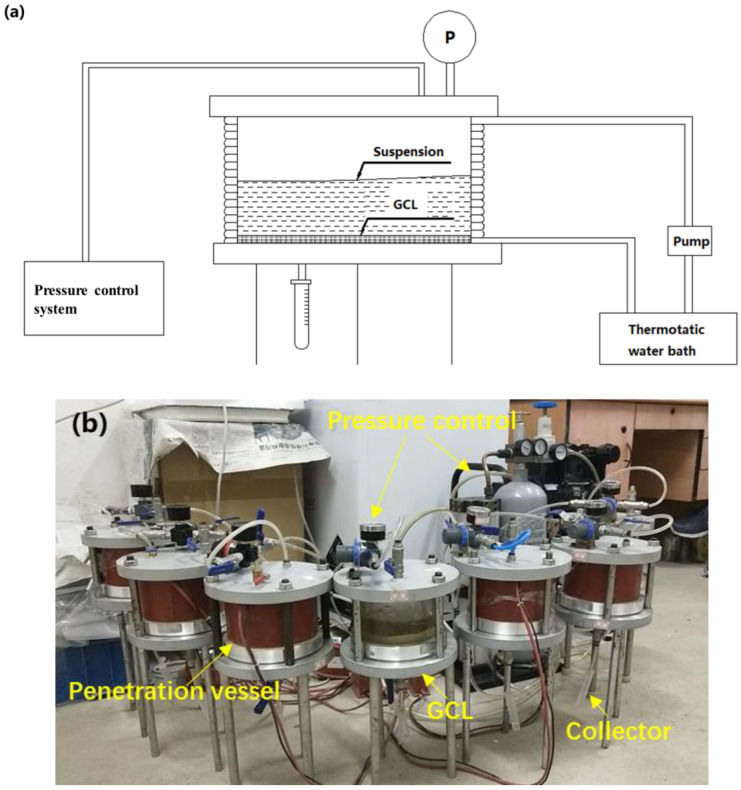
Experimental set-up: (**a**) schematic diagram and (**b**) modified flexible wall permeameter.

The transport process of GONPs in GCL was investigated using a self-designed apparatus. The apparatus was modified from a flexible wall permeameter [38]. The costumed-designed apparatus consisted of pressure control system, temperature control system (thermostatic water bath and peristaltic pump), penetration vessel and collector, as shown in Figure 2. Constant temperature and pressure should be maintained during the test. To maintain a constant pressure, a pressure gauge was used to monitor the pressure change in real time, and an air pump was used to adjust the pressure state. The temperature can be maintained through the indoor temperature control in previous studies. However, the temperature requirement for column tests in this work was pretty high. Therefore, water circulation was used to keep the constant temperature of the device. Water was heated by heating pump and injected into the flexible hose, which encircles the entire penetration device, as shown in Figure 2. The suspension was transported through GCL under the influence of gravity and specific pressure, which was determined according to the measuring value in LaoGang Landfill in Shanghai, China. The suspension passing through GCL successfully was collected by a collector.

### 2.5. Column Experiment

To investigate effects of temperature, IS and HA on the transport of GONPs in GCL, a series of column experiments were conducted. The detailed procedures were as follows:

(1) GCL was placed in the bottom of column. Deionized water (20 PVs) was injected into the column. The value of pore volumes (PVs) was determined by dividing effluent volume by GCL pore volume [33]. The deionized water was transported through the GCL and cleared the mini-impurity in GCL.
(2) NaCl crystal was dissolved in deionized water, and specific NaCl solution was mixed with GONP suspension to acquire GONP suspension with specific IS [33]. NaCl was chosen to control the IS because Na^+^ was the most-widespread and common positive ion in leachate. IS could be acquired through NaCl concentration and IS formula. IS value in each experiment is shown in Table 2.
(3) Specific content of HA was added into the deionized water, and the impurity of HA was removed using 0.45 μm filter membrane. Then, the HA solution was added into the GONP suspension to obtain the suspension with different HA content (Table 2).
(4) The GONP suspension with different IS and HA was injected into the column. The temperature was controlled by a temperature control system. To highlight the effect of single factor, the experimental scheme was designed based on simple variable method, as shown in Table 2.
(5) The initial concentration was 50 mg/L, and the pressure was 0.1 MPa in each column experiment. The values for temperature, IS and HA were determined based on previous studies, measured data and hydrogeological reports in many landfills [33].
(6) Effluent samples (1/3 PV each sample) were collected through automated collector until the volume of effluent exceeded 10 PV. The GONPs remaining in the GCL were washed by 6 PV deionized water.
(7) The concentration of GONPs in the effluents was measured through UV-vis.

## 3. Results and Discussion

### 3.1. Results of GONP Concentration Determination

**Figure 3:**
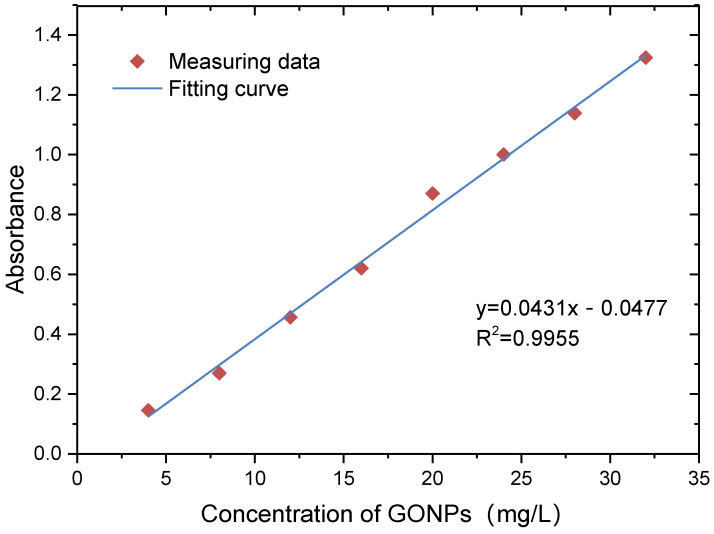
Relation between GONP concentration and absorbance.

Figure 3 shows that the absorbance (230 nm) increased with an increase in GONP concentration. The relationship between the absorbance of wavelength (230 nm) and the concentration of GONPs could be fitted through a linear equation, and the correlation coefficient was more than 0.99. This demonstrates that there was a good linear correlation between the GONP concentration and absorbance. Therefore, it is feasible to measure the concentration of GONPs through UV-vis in a specific concentration range.

### 3.2. Transport of GONPs in GCL

**Figure 4:**
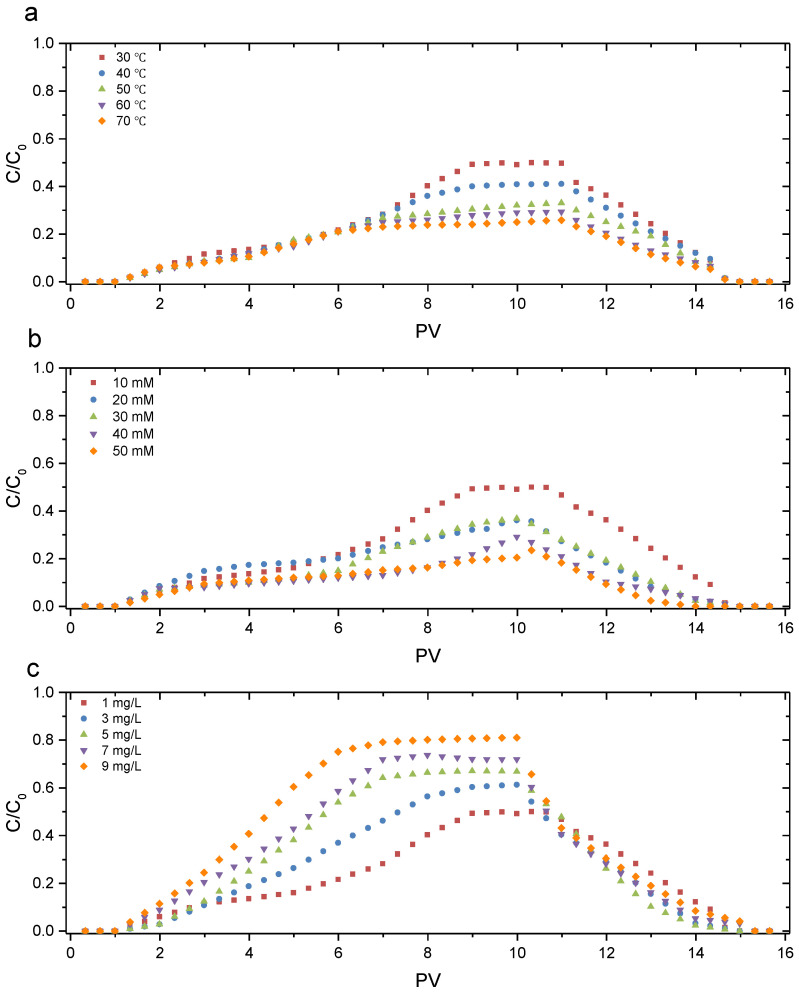
BTCs of GONPs under different conditions in GCL: (**a**) with different temperature, (**b**) with different IS and (**c**) with different HA.

We marked the concentration of GONPs before infiltrating the GCL as C_0_, and we marked that in effluents as C. The breakthrough curve (BTC) could be plotted through adopting PV as the abscissa and C/C_0_ as the ordinate [38]. The BTCs of the GONP transport in the GCL under different conditions are shown in Figure 4.

Figure 4 shows that GONPs could be transported through the GCL, but the mobility was different under different conditions. As shown in Figure 4a, the maximum value of C/C_0_ (C_max_) decreased from 0.49 to 0.25 when the temperature increased from 30 °C to 70 °C, indicating that a higher temperature hindered the transport of GONPs in the GCL. Previous studies have also reported a similar trend in the transport of GONPs in quartz sand [26]. This was because a higher temperature aggravated the Brownian movement of GONPs. The Brownian movement increased the aggregation of GONPs and reduced the stability of GONPs in suspension. Additionally, greater Brownian movement increased the collision probability between the GONPs and bentonite particles in the GCL [39]. As a result, a great number of GONPs were deposited on the surface of the bentonite particles, reducing the transport of GONPs.

It is also worth noting that the microbial decomposition in landfills will produce a lot of heat and promote an increase in temperature in landfills. Landfills should be kept at a higher temperature from the view of solely preventing leakage. However, a higher temperature will affect the microbial decomposition. Therefore, the inner temperature of landfills should be controlled at an appropriate value based on specific requirements.

The influence of IS on the BTC is shown in Figure 4b. The C_max_ was 0.5, 0.42, 0.37, 0.29 and 0.2 when the IS was 10 mM, 20 mM, 30 mM, 40 mM and 50 mM, respectively. The increasing velocity of C/C_0_ in the initial period also decreased with an increase in IS, suggesting that a higher IS inhibited the transport of GONPs in the GCL. This result is consistent with previous studies on the transport of GONPs in other porous materials.

The greater IS could inhibit the transport of GONPs for two reasons. Firstly, the electrostatic repulsion between nanoparticles and bentonite particles decreased with an increase in IS, promoting the aggregation and sediment of GONPs. Secondly, the electrostatic repulsion between the positive ion of the suspension and the positive ion of the diffusion layer increased with increasing IS. This resulted in the positive ions of the suspension entering into the adsorption layer, and the shrink of the bentonite double layer. Therefore, the flowing tunnel enlarged and the permeability coefficient of the GCL increased.

Figure 4c reflects the influence of HA on the BTC. The C_max_ increased from 0.5 to 0.8 when HA increased from 1 mg/L to 9 mg/L. Additionally, the increasing velocity of C/C_0_ in the initial period also increased with an increase in HA, suggesting that a more HA tended to be more favorable for the transport of GONPs in the GCL.

This was because HA covered the surface of GONPs and had an effect on the characteristics of the GONP surface. The HA covering the GONPs increased the repulsive force among the GONPs and promoted the suspending stability of GONPs. When the GONPs were transported through the GCL, the covering HA could increase the repulsive force between the nanoparticles and the bentonite particles, promoting the transport of GONPs.

### 3.3. Retention of GONPs in GCL

**Figure 5:**
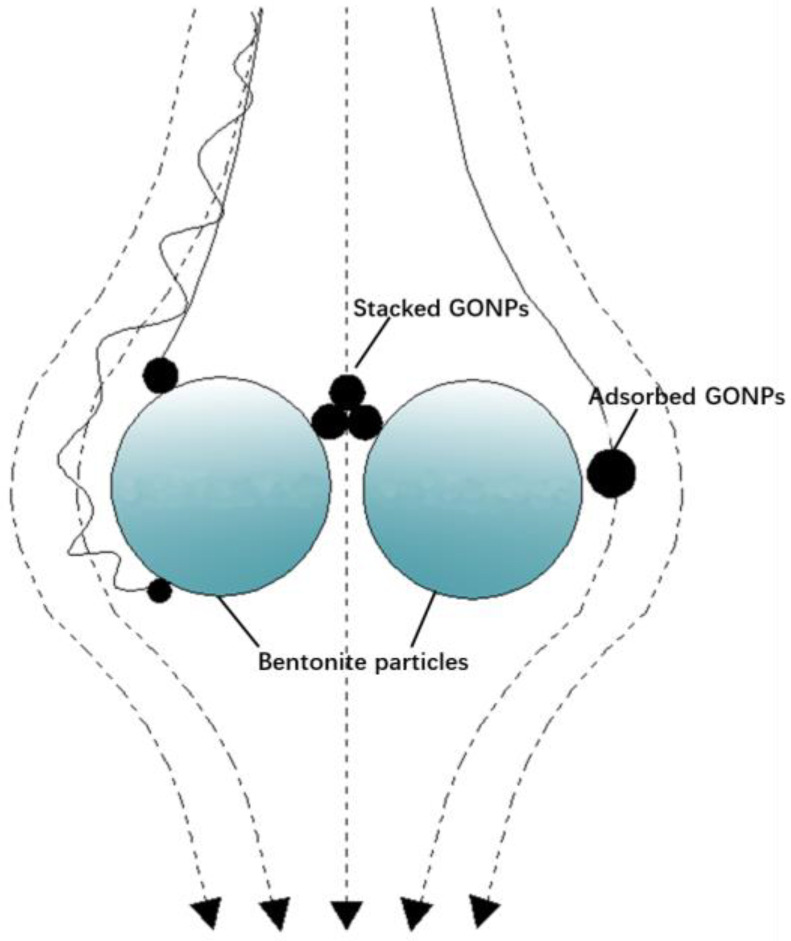
Schematic diagram of the deposition and sorption of GONPs.

The main components of bentonite (kaolinite, montmorillonite and illite) have been proven to have a strong adsorbing effect on GONPs [40]. Additionally, a small part of GONPs was found to deposit on the GCL after each column tests due to the low permeability of the GCL and the aggregation of GONPs (Figure 5). Therefore, the retention of GONPs in the GCL consisted of the adsorption of GONPs and the deposition of GONPs (Figure 5).

The GONPs that deposited on bentonite after each column test were collected and determined through UV-vis. The quantity of GONPs adsorbing on bentonite could be calculated according to the quantity of the total GONPs, GONPs in effluents and deposited GONPs.

**Figure 6:**
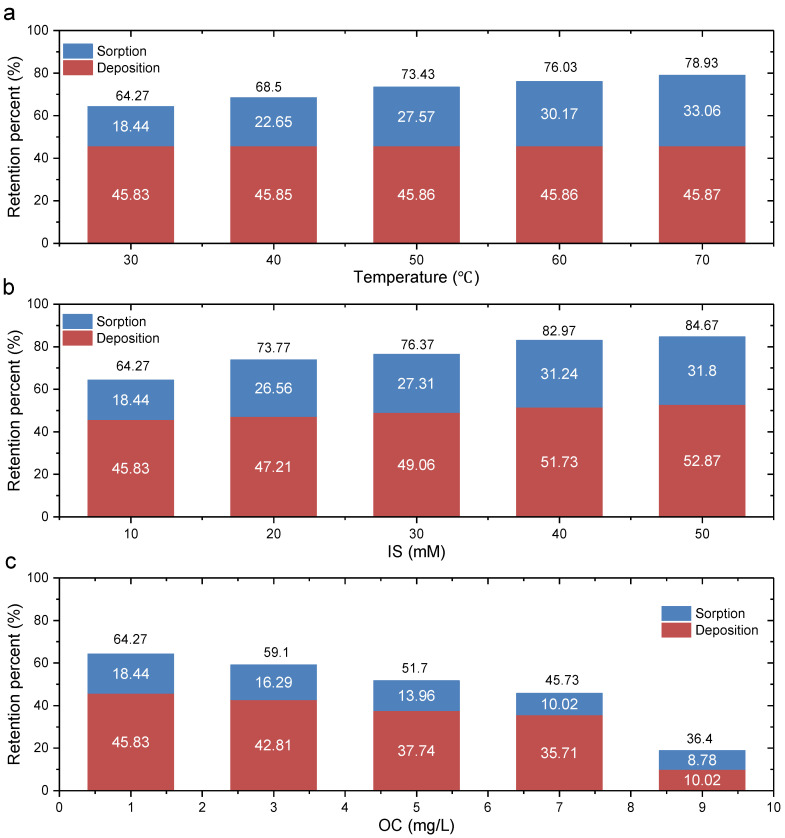
Retention percent under different conditions: (**a**) 30–70 °C, (**b**) 10–50 mM and (**c**) 1–9 mg/L.

The sorption ratio and deposition ratio of GONPs in the GCL under various conditions are shown in Figure 6. Figure 6 shows that the retention ratio of GONPs in the GCL exceeded 40% under different conditions (except Test 13), suggesting the high retention ability of the GCL.

The retention ratio (64.27–78.93%) and sorption ratio (18.44–33.06%) increased when the temperature increased from 30 °C to 70 °C (Figure 6a). This result is consistent with the experimental results of GONP transport in quartz sand and the BTCs in Figure 4. However, the deposition ratios under various temperatures were similar because a higher temperature could enhance the random Brownian movement and the collision frequency, but has little effect on the transport channel of bentonite particles.

When the IS increased from 10 mM to 50 mM, the retention ratio increased from 64.27% to 84.67% and the sorption ratio increased from 18.44% to 31.8% (Figure 6b). This was because the electrostatic repulsion between the GONPs decreased with an increase in IS. Additionally, the deposition ratio also increased from 45.83% to 52.87%, with an increase in IS because the IS could reduce the transport channel.

The retention ratio (64.27–36.4%) and the adsorption ratio (18.44–8.78%) decrease when HA increases from 1 mg/L to 9 mg/L (Figure 6c). A previous study has reported that HA was conducive to the mobility of GONPs because the addition of HA could result in stronger electrostatic repulsion and steric hindrance [41].

### 3.4. Comparison between Transport of GONPs in GCL and in Other Porous Materials

**Figure 7:**
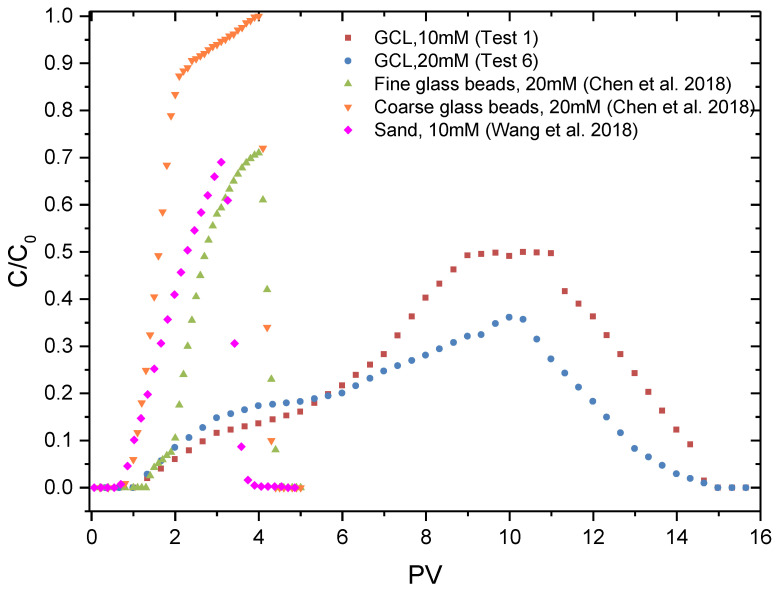
BTCs of GONPs in different porous materials [26,42].

To investigate the difference between the transport of GONPs in the GCL and in other porous materials, the BTCs of GONPs in the GCL (Test 1 and Test 6) were compared with the BTCs in fine glass beads (50–70 μm), coarse glass beads (600–850 μm) and quartz sand (0.425–0.5 mm), as shown in Figure 7. The BTC data in glass beads were provided by the investigation of Chen et al. [42], and the BTC data in quartz sand were provided by the results of Wang et al. [26]. The experiments were conducted under similar pressure, initial concentration and temperature.

Figure 7 shows that the curve shape of the BTC for the GONP transport in the GCL was quite different from the BTCs of the GONPs in other porous materials. The BTCs in quartz sand and glass beads were much steeper, indicating that the increasing rate of C/C_0_ was much greater. This was because the retention locations on the sand surface were of a limited number and filled up quickly over time due to Languirian dynamics blocking.

In contrast, although the GONPs could also be transported through the GCL, the shape of the BTC in the GCL was much smoother. The C_max_ in the GCL was much smaller than the C_max_ in glass beads and quartz sand and could be limited within a certain value. For example, the C_max_ was 0.71 (fine glass beads), 0.998 (coarse glass beads) and 0.69 (quartz sand), while the C_max_ in the GCL (20 mM) was only 0.36. More remarkably, the column test in the GCL was conducted under the condition that the HA of the suspension was 1 mg/L, and the C_max_ in the GCL will be smaller if there was no HA in suspension.

The transport velocity of the GONPs in glass beads and quartz sand was also much greater than that in the GCL (Figure 7) due to the strong adsorption of the bentonite particles. These facts proved that the retention ability of the GCL was much better than other porous media.

## 4. Limitations and Discussion

Some measures were taken to ensure the reliability of the test environment. A water cycle was used to keep the temperature of the penetration tests constant. Constant pressure was maintained through some devices such as pressure gauges and air pumps. However, the simulation tests still face many challenges such as accurately simulating landfill pressure conditions, effectively collecting effluent, maintaining seal integrity to prevent leaks, minimizing pulsation effects from the peristaltic pump, and ensuring the homogeneity of the nanoparticle suspension. Due to the limitation of the experimental conditions, these problems will be investigated in a further study.

The effects of some single factors such as temperature, IS and HA were investigated. However, the actual conditions of landfills are complex and the factors can influence each other. The inconsistency in peak BTCs suggests that the influence rule is more complex than the experimental setup can account for. The setup will be further improved, and the coupling effect of various factors will be studied.

## 5. Conclusions

The GONPs in landfills have great potential harm to the environment. To investigate the effects of temperature, IS and HA on the leakage of GONPs in the GCL, the experimental setup was modified and a series of column experiments were conducted.

(1) GONPs could be transported through the GCL, and the transport behaviors under various conditions were different: C_max_ decreased (0.49–0.25) with an increase in temperature (30–70 °C) because a higher temperature will aggravate the Brownian movement of GONPs; C_max_ decreased (0.49–0.20) with an increase in IS (10–50 MM) because a greater IS could reduce the electrostatic repulsion between nanoparticles and bentonite particles; C_max_ increased (0.49–0.8) with an increase in HA (1–9 mg/L) because HA covered the surface of GONPs and affected the characteristics of the GONP surface.
(2) Higher temperature, greater IS and lower HA will increase the adsorption ratio of GONPs. The deposition ratio increased with an increase in IS, and temperature had little effect on deposition ratio.
(3) The comparison between the transport of GONPs in the GCL and in other porous materials showed that the BTCs in the GCL were much smoother, and the retention ability of the GCL (0.36 C_max_) was much better than glass beads (0.71 C_max_) and quartz sand (0.69 C_max_).

## Figures and Tables

**Table 1:** Characteristics of GONPs.

Specific Surface Area (cm^2^/g)	Rate of Single Layer (%)	Thickness of Single Layer (nm)	Solubility (%)
1217	98%	1.0 nm	25%

**Table 2:** Experimental scheme of column tests.

	Test Number	Temperature (°C)	IS (mmol/L)	OC (mg/L)
Temperature group	1 (reference test)	30	10	1
	2	40	10	1
	3	50	10	1
	4	60	10	1
	5	70	10	1
IS group	1	30	10	1
	6	30	20	1
	7	30	30	1
	8	30	40	1
	9	30	50	1
OC group	1	30	10	1
	10	30	10	3
	11	30	10	5
	12	30	10	7
	13	30	10	9

## Data Availability

Data are contained within the article.

## Abbreviations

BTC	breakthrough curve
GCL	geosynthetic clay liner
GONPs	graphene oxide nanoparticles
IS	ionic strength
HA	humic acid
